# Participatory systems modeling in implementation research: Exploring benefits, facilitators, and future needs

**DOI:** 10.1017/cts.2026.10692

**Published:** 2026-02-04

**Authors:** Natalie Riva Smith, Jennifer L. Cruz, Jessica Gannon, Stephanie Mazzucca-Ragan

**Affiliations:** 1 Department of Health Policy and Management, School of Public Health, University of Pittsburgh, USA; 2 New York Academy of Medicine, USA; 3 Prevention Research Center, School of Public Health, Washington University in St Louis, USA

**Keywords:** Implementation science, systems science, complex systems, public health, participatory research

## Abstract

**Background:**

Implementation science increasingly uses participatory systems modeling (PSM) approaches to handle the complexity inherent to implementation science issues. To support the process of integrating PSM with implementation science, we aimed to understand and explicate the benefits, facilitators, and future needs of applying PSM to implementation research.

**Methods:**

We conducted semi-structured qualitative interviews with 23 researchers (n = 18) and practitioners (n = 5). We purposively sampled participants and identified additional participants through recommendations. Interviews were inductively analyzed. Key concepts were identified via iterative description, comparison, and conceptualization.

**Results:**

Engagement with people in the system was typically focused in earlier stages of PSM approaches, while engagement with decision makers occurred throughout a project. PSM approaches benefited researchers (e.g., improving the relevance of research) and practitioners (e.g., promoting systems thinking). Both benefited from the visual, intuitive nature of PSM and the ability of PSM to reflect partners’ input transparently. Facilitators included trusting relationships and conducting practice-driven research. Participants emphasized the need to improve funding opportunities for engagement and increase training in systems modeling facilitation.

**Conclusions:**

Our findings can help move the field towards fully partnered and impactful implementation research that addresses the systems problems. While PSM approaches are promising, if not done according to best practices of partnered research, they will reproduce existing power imbalances and consultative engagement patterns between community partners and academics.

## Introduction

The multidisciplinary field of implementation science focuses on improving the translation of research findings and evidence-based interventions into public health and clinical practice [1]. Major areas of study in the field include understanding key determinants of implementation [2,3]; developing, testing, and selecting strategies to promote implementation [4–6]; and measuring implementation outcomes [7,8]. The field also emphasizes participatory research, defined as research that engages implementation practitioners (e.g., clinic providers, community members), particularly when the goal is to address health inequities within entrenched systems [9–11].

Engagement in participatory research can range from consultative, where partners are engaged in well-defined parts of the research, to partner-led, where partners set the agenda and researchers support the project [10,12]. However, participatory research is still not the norm, and the field has struggled with the meaningful engagement of partners in research, in part because of the time and resource investment required to build and maintain partnerships [10,12,13]. These difficulties are not unique to implementation science and have been noted in broader public health and clinical research [14,15]. As the use of participatory methods becomes more frequent in implementation science, it is important that the field works to avoid performative, tokenistic partnerships that retrench existing systems and power dynamics [10].

In addition to wrestling with meaningful engagement, implementation science must also contend with dynamic complexity [16–20]. Key elements of complexity encountered in implementation science include heterogeneity in resources or goals among implementation actors, interdependence among those actors, and feedback loops between actions [16,17,19,21]. Recognizing the need for approaches that can manage this complexity, scholars have called for implementation science research to integrate methods from the field of systems science [17–19,22]. Systems science, with its focus on understanding the workings of complex systems, provides a toolkit for conceptualizing, making sense of, and embracing the complexity within implementation settings [17–19,23–25].

Like implementation science, systems science also emphasizes participatory approaches, or “participatory systems modeling” (PSM) [21]. PSM approaches to systems science research are broad and applications can range from developing visual representations of systems to formalized computer models of systems [21]. Like participatory implementation research, applications of PSM can also vary by the level of partner engagement. One PSM approach, community-based system dynamics, has a major focus on deep engagement and building capacity in communities to understand systems and advocate for effective systems change [26]. Reviews of different PSM methods have suggested that participation strengthens individual insights and commitment to conclusions, and group communication quality and consensus [27–29]. However, achieving increased capacity, empowerment, and other benefits takes significant work and resources, including the time to develop relationships with groups and communities and developing the skills required to facilitate these processes [26].

Work applying PSM to implementation science research is growing. For example, Cruden *et al.* show how a method called group model building can be applied to operationalize implementation strategies in the context of making decisions about child maltreatment prevention interventions and implementation in local contexts [30]. Kasman *et al.* engaged state health department officials to support the development of an agent-based model which was used to understand how different organizational changes might impact rates of mis-implementation in health departments [31]. Other work has been more methodological and introduced specific approaches to apply PSM to implementation mapping [21,32], examine implementation mechanisms using systems science [33], and use PSM as an implementation strategy [34]. This methodological work is promising, as a previous review examining studies that combined systems science and implementation science noted many opportunities to develop guidance on how exactly these fields should be integrated, including clear definitions of “systems” and guidance on how implementation science constructs could be adapted to systems work [35].

This new line of work represents a major step forward for transdisciplinary health research and brings up important process questions that we contend should be interrogated alongside the increased development and use of these methods. Do PSM approaches recreate the patterns of consultative engagement identified in participatory implementation science, and participatory work more broadly? Are implementation science researchers seeing the benefits of PSM that we would expect? What is working well in integrating these fields? And how can we move forward most effectively? To our knowledge, no study has empirically investigated such questions by considering how researchers and practitioners perceive the application of PSM approaches to implementation science research. In this paper, we seek to fill this gap by conducting qualitative interviews with researchers and practitioners to examine perceived benefits, facilitators, and paths forward when applying PSM to implementation science research.

## Methods

### Study participants

We conducted semi-structured qualitative interviews with researchers and practitioners engaged in PSM for implementation research and practice. We considered researchers any individual working at an academic institution, while practitioners were individuals whose primary role was not in academia or research-focused. Our initial list of participants was developed using our knowledge of individuals who had conducted work using PSM and implementation science (derived from publications and our professional networks). We were intentionally inclusive of all PSM projects regardless of their depth of engagement, as we were particularly interested in understanding typical levels of engagement. We purposively sampled participants across career stage, institution, and applications of PSM and invited participants via email. Additional participants were recruited via recommendations from purposively sampled participants, which helped to ensure that we had identified key researchers in the field and practitioners through their existing relationships to study participants. We were particularly interested in interviewing the practitioners who had worked with the researchers we interviewed, but researchers were often hesitant to refer us to their partners due to concerns about overburdening them or their ability to answer our questions. One invited participant actively declined (researcher) and five did not respond to our invitation (one practitioner, four researchers). A consent overview was emailed with invitations, and verbal consent was obtained by the researcher conducting the interview before recording. All participants were offered a $50 gift certificate.

We also asked participants to complete a short questionnaire to provide context for our results (full questionnaire in the supplementary materials). We collected standard demographic information (age, self-reported gender, self-classified race, self-classified Latino or Hispanic ethnicity) and professional information (education, area of study, years in the field, and systems science methods used). We also asked participants to use a slider scale and report the degree of practice focus of their work (0 = completely research-focused, 100 = completely practice-focused). We conducted 24 interviews with 23 individuals (one individual interviewed twice due to time constraints), and descriptive information is included in Table 1. Of the 23 individuals, 5 were classified by the study team as primarily practitioners and the other 18 were primarily researchers. Here, 22 out of 23 individuals responded to the contextual survey (Table 1). Participants had a median age of 43 years old (range: 33 to 65) and self-identified primarily as female (*n* = 16, 73%) and white (*n* = 16, 73%). No participants reported being of Latino or Hispanic ethnicity.

**Table 1.** Descriptive information on interview participants (22 participants)

Variable	Median (Min, Max)	N (Percent)
Age	43 (33, 65)	
Female		16 (72.7%)
Self-reported race		
White		16 (72.7%)
Asian		5 (22.7%)
Another group		1 (4.5%)
Not of Latino or Hispanic ethnicity		22 (100%)
PhD		19 (86.4%)
Self-rated research/practice*	36 (0, 100)	
Years in field	15.5 (3, 40)	
Methods used by participants		
Social network analysis		16 (72.7%)
Causal loop diagraming (inclusive of group model building efforts)		16 (72.7%)
System dynamics		11 (50%)
Agent-based modeling		15 (68.2%)

*Notes: 0=completely research-focused, 100=completely practice-focused.

### Data collection

Our interview guide was developed based on our key research questions about the benefits, facilitators, and future needs in applying PSM to implementation research (see supplementary materials). To ground our discussion, we asked participants to talk about one of their most recent PSM projects. This helped ensure that participants remembered details and provided a starting point for discussion. After describing details of the project, we asked participants to reflect on the benefits of the participatory nature of the project, the benefits of the systems science nature of the project, and the benefits of integrating participatory research, systems science, and implementation research. We specifically probed for details about the potential benefits to both researchers and practitioners. Our interview guide also included questions about the difficulties of engaging in PSM work, changes they might make to their own approach in future projects, and what is needed to move the use of PSM for implementation research forward.

### Data analysis

The team met regularly to discuss interview progress and assess thematic saturation. One-hour interviews were conducted by the first author during their postdoctoral fellowship (NRS) and recorded via Zoom and transcribed using an online service (Otter.ai) for analysis. Our initial codebook was created from our interview guide and revised as we iteratively coded interviews in NVivo moving from deductive to inductive coding using a reflexive thematic approach (final codebook available in supplementary materials) [36]. All interviews were double-coded by members of the research team (NRS, JLC, JG, SMR) who met weekly to resolve discrepancies via consensus, discuss, and reflect on findings [37]. We then iteratively synthesized coded data to generate results, reviewing coded data in detail and inductively documenting key concepts that repeatedly emerged via iterative description, comparison, and conceptualization [37].

Throughout this iterative process, the team refined the codebook as new concepts were identified and revisited earlier transcripts to ensure consistent application of codes. We also updated our interview guide in response to preliminary analyses, which allowed us to probe emerging ideas in later interviews. Once coding consistency was established across coders and no new codes or themes were identified, we determined that thematic saturation had been reached. We also considered the information power of our sample to be sufficient to address our research aim, given the specificity of our study focus (benefits, facilitators, and needs in participatory systems modeling), the relevance and expertise of participants, the strong quality of dialog, and the cross-case analytic approach – factors known to enhance adequacy of smaller qualitative samples [38]. As part of our reflexive approach, we reviewed discrepancies and examined the reasons underlying our initial interpretations. We deliberately compared perspectives within the team to ensure that no single viewpoint dominated analytic decisions. To promote transparency, we maintained an audit trail of code definitions, revisions to the codebook, decisions made during theme development, and the process for resolving disagreements.

### Team composition

The team’s training is interdisciplinary, with backgrounds in biostatistics, health policy, decision science, social epidemiology, behavioral sciences, social work, nutrition, intervention development, and implementation science. We are early-career professionals with a varying range of experience participating in PSM projects including group model building, network analysis, and agent-based modeling, and have varying levels of expertise in participatory approaches. Our motivation to pursue this research project arose out of our ongoing training and learning about PSM and its applications within implementation science, particularly to ensure that these methods are rigorously applied in implementation research.

The Harvard Longwood and Washington University in St. Louis deemed this study exempt.

## Results

### Project description

Interviewees described a range of implementation-focused PSM projects: visually mapping complex systems or understanding network structures (specifically related to implementation), identifying and eliciting parameter values for simulation, obtaining needed data for simulation, understanding possible intervention or policy options within a system, improving the products that arose from systems science work, improving how users interpreted systems model results, providing decision making support, and planning for sustainability.

The individuals engaged in these PSM research projects (“partners”) were, broadly, either people in the system being studied (i.e., community members experiencing food insecurity, implementation providers/practitioners) or decision makers (i.e., public health department program managers, school board members). We observed a pattern between the step/phase of a project and what partners were engaged. Typically, people within the system were engaged as researchers mapped the system or network structures. On the other hand, decision makers were partners across the project life cycle, from helping to map/understand the system, to identifying leverage points or decision/policy options, to interpreting results and using results to drive decision making.

**Figure 1.**
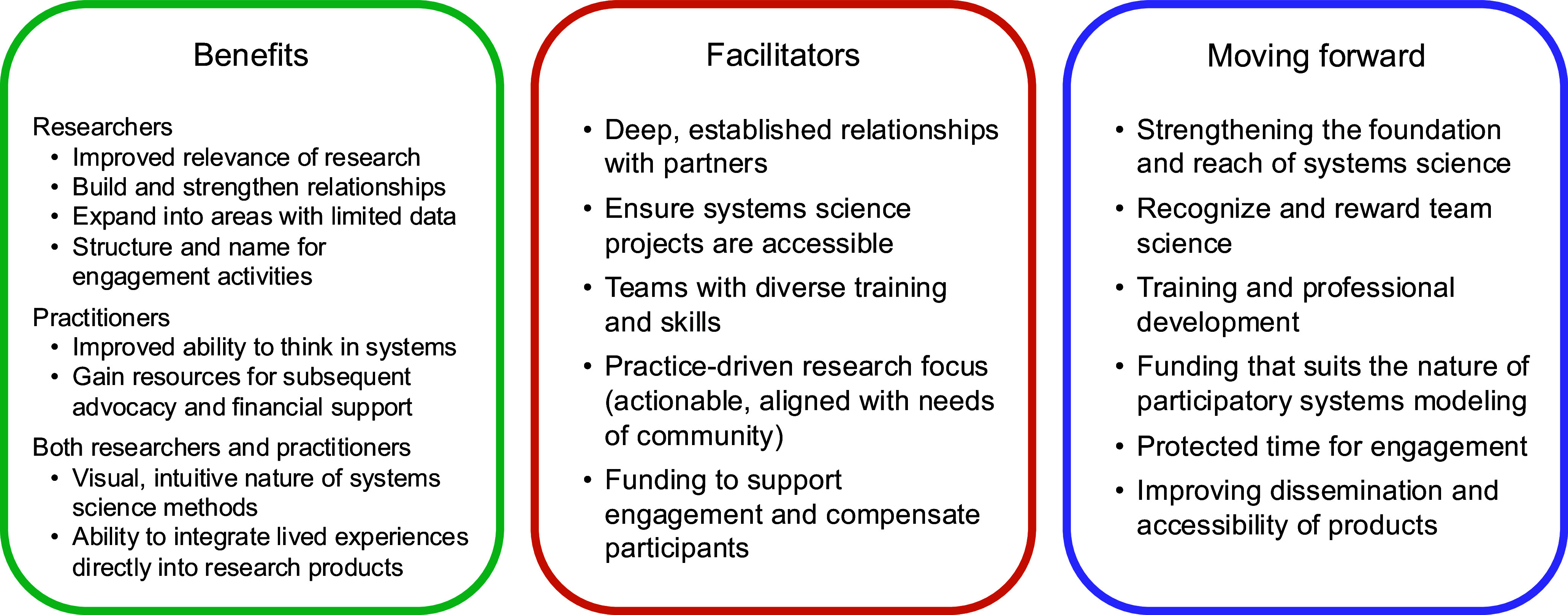
Summary of analytic results.

Figure 1 provides an overview of the benefits, facilitators, and future needs identified by participants. Representative quotes for our results are shown in Table 2.

**Table 2.** Participant quotes

Theme	Illustrative Quote(s)
**Benefits of using participatory systems science methods in implementation science to researchers**	
Improved relevance of research	“…like a lot of participatory or different types of community engaged stuff, it helps you like, know what not to waste your time on. Like, you might think something’s this really great idea or this thing that it’s like, well, the literature says, these are the best interventions and let it out. And people are like, No. And so it kind of does can help you hone in on that and what they need.” (Participant U) “…getting to the bottom of the problem, is actually the biggest problem is the need for the simulation model that we have. And we only learned that through engaging people who are misusing opioids. So that I would say the models are becoming a lot more realistic.” (Participant L) “You can be talking with them and say, ‘how would you use this?’ You know, ‘would you actually ever present any of this at a city council meeting?’ Or ‘would you actually ever put this if you’re talking, let’s say to CDC, would you actually ever put this in your RFAs for the states?’ And one, you’re getting them to think about that, but also, you’re getting them to tell you, Oh, they actually might put this in the RFA. So let’s make sure that what we’re building is going to be relevant for that.” (Participant M)
Build and strengthen relationships	“So it [engaging partners in modeling] definitely spurred I think, bigger discussions that don’t ever happen, because it’s not in the data.” (Participant D)
Expand into areas with limited data	“…because … looking at things where you have a limited amount of data, you know, there’s going to be a variation about all the different parameters that you’re not going to have a point estimate of anything. But you’re going to have some idea of how often it happens.” (Participant C)
Structure and name for engagement activities	“… the NIH will fund you to do it, right. I mean, like, you get publication out of it, you get grants out of it, instead of just being like, we work with community partners, we did consensus building, … but if you start putting these, like sort of more technical terms on, but it’s really the same type of process, you know, it’s just so that’s a little bit like, it’s a little bit like pessimistic, but it’s true.” (Participant T)
**Benefits of using participatory systems science methods in implementation science to practitioners**	
Improved ability to think in systems	“Yeah, no, we did some things [before partnering with researchers to use systems science]. But it was too this is what we need the overall picture, because this can lead us on for years. We can work on this for a long time… running around like, a chicken with their head cut off…, but this gives us a goal to work to an overall, you know, picture, and this is what we were missing.” (Participant O) “…a difference would be that it helps people really intentionally think about the consequences of what they’re proposing. And this could be good or bad consequences.” (Participant D) “they’re considering their own their own things that they want to accomplish based on others in their community, so that they’re not duplicating effort, they’re hopefully addressing gaps that already exist. And if there are others doing work, rather than duplicating they can work together to hopefully have a better chance of having an impact, because they’re joining together to do that.” (Participant I)
Gain resources for subsequent advocacy and financial support	“…some of these knowledge translation documents that we’ve built… based on some of these dashboards were used in ***** in the city council meeting to help pass stronger tobacco control legislation.” (Participant M) “People actually ended up copying and pasting some of the visualizations into their grant applications to funders, to potential funders and into reports to existing funders. And that was that was interesting. I hadn’t anticipated that people would use it for that. But people did.” (Participant G)
**Common benefits to researchers and practitioners**	
Visual, intuitive nature of systems science methods	“…one nice thing about agent-based modeling and like thinking in those terms is it does, it’s naturally very intuitive to a practitioner, because they are dealing with someone’s immediate decisions and behavior. And so our partners are really excited about that part of it.” (Participant H) “We didn’t know how to put it in on paper or in graphical format, or even an order. So the benefit of having that [causal loop diagram], and being able to look at it, and follow it is pretty amazing. Because it’s, it’s not so jumbled up in your head, you know?” (Participant O) “I think that agent based modeling offers a lot of advantages for engaging with stakeholders, because it can be very visual, because it’s role based, because you can usually explain what’s going on in and even in quite a sophisticated agent based modeling without showing any math, no P value tables, no equation and you can tell stories about where you personified individuals who you’re following through the model and then all of my work I take full advantage of that capacity and agent based modeling to engage with stakeholders.” (Participant K)
Ability to integrate lived experiences directly into research products	“… there are opportunities, especially if you’re careful about how you structure like explaining what the models are, what you mean by inputs, … where you really show them, this is where like your direct input, like directly informs what emerges. That’s… a nice touch.” (Participant N) “…And then later, when they were looking at the map [can] be like, ‘Oh, I literally told you that story. And like, it’s right here.’ And that happened, like multiple times. And I mean, they maybe didn’t realize that they weren’t the only people who told us that story. But they were like, oh, that’s what I said. And we’re like, yeah, we listened to you. So that was powerful.” (Participant F)
**Facilitators to using participatory systems science methods in implementation science**	
Established, deep relationships with partners	“…because of that trust, and that long standing relationship, … we’re very transparent, and we try as much as we can to distribute funds as equitably as we can, as each different type of grant mechanism will allow. So like, I think we’ve gotten to a point where the community partners trust us, they know we live the grants life, they know that a lot of the support we provide to them and what we’re able to do is only like, driven by, like our ability to successfully get grants that then drive the work. And so I don’t think there needed to be like, like, let’s sell you on the system science piece, they trust us methodologically” (Participant N)
Actively make systems science projects accessible	“But the idea is, is to visually kind of get across lots and lots of complex things that are happening in hard math behind the scenes in a really user friendly, intuitive way. So the more successful you are at that the more capable you are communicating, hey, we’ve built up this model that captures all these relevant aspects of the way that the world actually works, [and it] lends credence when you when you finally show your results again… You know, people are gonna want to know what’s going on behind your model, going on behind the scenes in your model. And if they’re, they’re kind of shown just the hard numbers and the equations, I found that a lot of people kind of, they’ll back away, they’ll kind of feel kind of bullied away, almost.” (Participant J) “By having that visual, they can immediately sort of jump into and understand what you’re thinking about, and immediately start to contribute. So I, I think, maybe the diagram like, necessarily gets at, like, all of the complexities involved… And I think people are, like, often, like, even surprised, like pleasantly surprised by their own ability to like, really jump into that fast, … and that’s both like patients and providers and other types of stakeholders.” (Participant P)
Teams with diverse training and skills	“Think about it as like a research skill and have somebody on their team that has that skill. Because it’s kind of like qualitative work. Everyone thinks that you can just do it. Yeah, I mean, you can physically, [but] it’s not gonna be a good idea, which [emphasizes] that there is a knowledge base and there is a skill set. And so just to have, if you’re not that person, have somebody on your team who knows this world, because it’s not just like a tack on.” (Participant Q)
Practice-driven research focus	“From a research perspective, like maybe generalizability people worry about, like whether or not there’s an influence on, you know, is this truly objective research and that sort of thing. But then we also would possibly argue that we’re getting more people to participate in the work of informing, because we’re removing some of those barriers.” (Participant E)
Funding to support engagement and compensate participants	“We set up a table and we like make sure that people get something, like are well compensated, but not to a point where it feels coercive that they, you know, sit with us… Well, I think the things we definitely need to work on are the how to roll it [project outputs] back out to them, how to share back with them how to not just extract but also you know, change something for them.” (Participant E)
**Future directions to improve the use of participatory systems science in implementation science**	
Strengthen the foundation and reach of systems science	“I think it would be helpful to have some… thoughtful people thinking about like, where does the engaged systems pieces fit into… the existing frameworks that are like, thinking about, like implementation… [or] public health practice. I kind of see those overlapping often… So there’s like the frameworks for like, how you do implementation, how you do in your basic public health practice, strategic planning kind of work, like I think, some thought into, right, engagement with these systems pieces. Where does that fit into those frameworks?” (Participant A)
Recognize and reward team science	“The main thing that would help the most is interdisciplinary teams or team science. At least in my experience, I rarely see one individual who is capable of truly engaging with communities and good at-and can do research and has the technical expertise to do systems science modeling… three really different skill sets, and it helps tremendously if you can have people who can serve in and are really robust in those fields in and of themselves and not be expected to do all of the things” (Participant H)
Training and professional development	“I think the problem right now is that, like, I was trying to get into participatory group model building and trying to find someone who could actually do that. There’s like, less than 20 people around the country who actually do it. Right. So there’s just a lack. There’s no, there’s no pipeline… Now that there’s a lot of people that do CBPR [community-based participatory research], that’s the value that could potentially, you know, see the value in system science approaches. And then there’s like a ton of like, engineer trained system science modelers that are incredibly skilled, but like, the people that are in that overlapping Venn diagram are like, there’s like five people in the country. You know, and so there’s, there’s like a pipeline problem.” (Participant T)
Funding that suits the nature of participatory systems science work	“And that’s hard to get through NIH, because reviewers want to know that you have all the data that you need. And that, you know, you have that you know how to do this project. And I don’t want to structure the model fully until I have had all the conversations, you know, in the community to be able to do it. So NIH is hard for this type of work” (Participant B)
Protected time for engaging community partners	“You need this time in this space to really build those relationships, build a track record, think about how you want to, you know, facilitate all of this, be able to show people what it looks like” (Participant B)
Improving dissemination and accessibility of products	“…making the outputs more accessible. And sharing findings…[through] really cool, user friendly, interactive tools that you can put up on the web, like things like that. There is probably a shortage of people who can create those things.” (Participant W)

### Benefits of using PSM in implementation science

#### Benefits to researchers

Interviewees reflected how conducting PSM work *improved the relevance of their implementation research* across the full research process, from the development of an idea to the dissemination of findings. First, partnered research helped researchers think about the problem they were focusing on and the questions they were asking. Partnering with people with relevant lived experiences also helped provide a more realistic understanding of the system and models. Third, research products were more actionable and useful when they incorporated input from partners. We considered each of these a distinct opportunity for benefit but note that they are quite interrelated: for example, partners clarifying the focus of a research project are key to making the model more realistic, which in turns helps to improve the usefulness of the model for driving change.

A second benefit discussed in interviews was the way that PSM research helped to *build and strengthen relationships*. Sometimes the projects helped to build new relationships which led to further collaborative efforts or helped strengthen existing relationships by spurring deeper discussion. For example, convening folks in a room and using systems science methods was noted as an important tool to facilitating conversations among organizations or individuals that might not otherwise talk.

One interviewee mentioned that a key benefit to researchers is that PSM allowed their team to *expand into an area that had very limited data*. By engaging partners, they were able to understand the structure of the problem, begin to put rough estimates on parameters, and understand what types of data should be collected in future work. Another important benefit noted by one interviewee was that participatory systems science is one way to *put a name on the work that is being done with partners* and “get credit” for engagement within the expectations of their academic appointment and incentive structures.

#### Benefits to practitioners

Interviewees discussed how participatory systems science helped *improve practitioners’ ability to think in systems*. Two key elements of systems thinking emerged as aspects that were particularly important to practitioners. First, engaging in systems science projects helped practitioners identify future goals and more effectively think about the long-term plans for their community or organization, and better consider the long-term consequences of how system changes might manifest. Practitioners also noted an improved understanding and awareness of interconnectedness within a system. Several interviewees discussed how using systems science methods improved implementers’ understanding and awareness of their network and the other actors that exist within that network, sometimes leading to additional connections that they might not have known before or helping to avoid duplication of efforts.

A second benefit specific to practitioners was that participating in PSM projects helped them gain resources to structure advocacy discussions and/or garner financial support from other institutions. PSM products – such as model visualizations or predictions – helped support advocacy to policymakers by providing tangible products that captured people’s voices and lived experiences and that showed the potential impact of a change within the system. Beyond advocacy, PSM products were also used to help support practitioners’ grant proposals for further funding.

#### Benefits to both researchers and practitioners

Two aspects of PSM projects were highlighted as beneficial to both implementation researchers and practitioners. First, the *visual, intuitive nature of systems science* was discussed by both parties. Two researchers discussed that systems science felt more intuitive to their partners than traditional statistical methods, making it easier to engage with and making the end results something that people can act on. Conversely, practitioners could more easily see the relationships that were being modeled and noted that it helped them get into deeper conversations more quickly.

The visual nature of PSM methods was also an important way to reflect participants’ perspectives back to them and show that they were being listened to, which overlaps with another key benefit that both researchers and practitioners noted: *the ability of systems science methods to integrate lived experiences and input directly into research products*. This helped to build trust and relationships between researchers and practice partners.

### Facilitators to using PSM in implementation research

Interviewees reflected on a variety of factors that were both barriers and facilitators to achieving the stated benefits of these methods. Here, we present facilitators only to streamline the discussion. A key facilitator highlighted in many interviews was having *established deep relationships with partners*. These types of relationships were seen as having the trust necessary to effectively work through these projects, which are time intensive and require commitment and resources from partners. High levels of trust were also helpful to ensure that partners did not view research as extractive as there was an understanding of the methods and research processes that researchers need to follow. It was also mentioned frequently that academic researchers needed to show up for partners by coming to them, building trust, and meeting their needs to develop and maintain these types of relationships, particularly with high turnover rates among partner organizations.

Interviewees also emphasized the importance of *actively making systems science projects accessible* to everyone involved via capacity building opportunities and accessible communication so that partners from various audiences (e.g., community members, decisions makers, policy makers) can understand and benefit from the knowledge and outputs produced in the work. Interviews highlighted the need for capacity building around systems science approaches among partners to facilitate a deeper understanding of the methods used for a PSM project, and to help partners potentially use systems science methods in the future, in their own everyday work (aligned with the benefit of improving practitioners’ ability to think in systems). Making sure that information was delivered in a non-technical manner and utilizing visuals to convey modeling outcomes were provided as examples by several respondents. Taking these approaches were described as entry points for more robust discussion and involvement from all partners in PSM projects. Visual approaches were also highlighted as ways to make projects accessible and sustain engagement from partners throughout the project.

Having a *team with a diverse range of training and skills* was highlighted as a major facilitator. Primary examples of areas of expertise needed on a team conducting PSM research were community engaged research, systems science research approaches and methodologies, and technical model building skills. It was discussed that taking a team science approach was especially necessary for participatory systems science research as very few individuals possess all the previously mentioned training and skill sets. In addition to researchers with these skills sets being present on the research team, it was also highlighted by in one interview that having a partner serve as a liaison to the research team is a critical way to help build trust between researchers and community members.

Interviewees emphasized that *the goal of PSM research should be practice-driven*. Practice-driven research is research that is tethered to the lived experiences of implementation practitioners [39]. Participants described their conceptualization as conducting research that is actionable and addresses the immediate needs of partners to avoid simply extracting information from communities. This was highlighted as a facilitator particularly as it aids in securing buy-in from partners. Focusing on practice-driven work also helps with continued engagement by providing a clear understanding of the overall benefit of the project to partners and larger communities. An important aspect of this facilitator that was highlighted by one interviewee was ensuring that researchers are transparent with partners regarding their research goals and compensation of participants. This allows partners to set their expectations and have a clear understanding of what the end goals of the project are but requires flexibility that is in tension with typical academic funding mechanisms that require methodological decisions to be made a priori.

Finally, *sustained funding for PSM work* was also a critical facilitator. This was consistently discussed in relation to the duration of engaged systems science projects and the need to adequately compensate community members for their expertise.

### Future directions and needs

Interviewees provided considerations to improve the use of PSM in implementation science. First, a major need that emerged was *strengthening the foundations and reach of systems science*. One possibility discussed was developing a common framework and language for talking about systems science. This unity could help researchers and practitioners more easily communicate with each other and accelerate progress in the field, such as understanding where community engagement, systems thinking, various systems science methods, and implementation science/practice intersect. Also, interviewees highlighted a need for an *increased awareness of systems science thinking and methods* among the broader research and practice communities. This discussion was primarily in the context of sharing these methods with researchers and practitioners who are not yet trained in or experts in systems science methods. Specifically, there was a discussion of awareness related to the foundational thinking within the field, the tools available to systems scientists, the complexities of systems science, and the accessibility of the methods to individuals.

Interviewees also discussed the need to *recognize and reward team science*, given its major role in PSM projects. Reflecting both this and the importance of building out the use of these methods, participants also highlighted the importance of *training and professional development* opportunities to increase the numbers of individuals who are sufficiently skilled to lead high-quality PSM research. PSM research applied to implementation science draws on systems science, participatory research approaches, and implementation science. Individuals leading or participating in PSM projects do not need to be experts in all fields but must understand how to integrate the fields. Interviewees noted that individuals with the intersection of these skills are rare, making team science an even more important aspect of these projects.

Interviewees discussed a lack of *available funding that suits the nature of PSM work*, related to the discussion of funding and practice-driven research as facilitators. Interviewees noted that there is somewhat of a mismatch between the typical funding mechanisms of public health and clinical research and how PSM research requires being responsive to partner needs, i.e., practice-driven. There are many unknowns when proposing a larger PSM project, as early stages of PSM projects often involve understanding the system and identifying key areas of focus or intervention. Funding agencies and reviewers are often uncomfortable with these unknowns in proposal applications.

Interviewees also discussed the need for *protected time to build relationships* (logistically and financially) to support PSM projects. It takes time to build relationships with partners and to demonstrate trustworthiness as a researcher. Then it takes additional time to work with partners to explain and plan for a PSM project. Interviewees underscored that doing this requires having time that is protected and funded for this explicit purpose.

Last, interviewees noted the need for improving the dissemination of PSM products. The products of PSM projects should be useful, i.e., they should be actionable or provide some kind of solution, and they should be tailored for the end user receiving the product. Interviews discussed a need to think about “how do you make models useful” to different end users, and a need to find people who can translate PSM research products to partners.

## Discussion

Through discussions with researchers and practitioners experienced in the use of PSM methods, we explicated many benefits of using PSM. For researchers, benefits included improving the relevance of their research, building and strengthening relationships with partners, and expanding into new research areas. For practitioners, benefits included the improved ability to think in systems and access to resources that can improve advocacy efforts. These benefits to practitioners are similar to the benefits of specific PSM approaches: group model building can improve individual insight and commitment to conclusions [28,29] and a core principle of community-based system dynamics is building capacity for community advocacy [26]. Benefits also align with acknowledged benefits of community-based participatory research, including strengthening community capacity, developing more contextually appropriate and effective interventions, and increasing the overall validity of research methods [15,40].

We also identified novel benefits of PSM methods relevant to both researchers and practitioners: the visual, intuitive nature of PSM methods and the ability of these methods to clearly reflect partner input. These benefits improve participatory research’s accessibility (partners don’t need significant training to understand visuals of the system) and transparency (partners can directly see where their input has been included).

We argue that these benefits provide evidence that PSM can be particularly useful for implementation science research. Using PSM to expand into areas with limited research is particularly relevant to implementation science: approaches acknowledging complexity and using systems thinking can illuminate core uncertainties and guide future data collection [16,41]. In addition, because we have identified that PSM projects have benefits to both researchers and practitioners, applying these methods in implementation science can help begin to move the field away from the consultative nature of most participatory implementation science research [13]. For example, PSM methods provide a way to integrate and reflect partner input directly into models of implementation processes, and strengthen relationships with implementation partners. However, this argument depends on PSM approaches themselves being rooted in robust relationships between researchers and partners and not falling into extractive engagement practices.

Reinforcing this concern, we observed a dichotomy in how researchers talked about the who and why of engagement. Most often, people within the system (e.g., community members) were engaged as “people with lived experience” to help researchers understand system structure in the early stages of the project. Conversely, people engaged as “decision makers” were engaged across the full project life cycle, helping to identify leverage points for change or interpret results. This pattern suggests a need to consider how we engage with “people with lived experiences” in PSM to avoid reinforcing existing patterns of consultative engagement observed in implementation science that PSM could disrupt [13]. This echoes the focus within community-based system dynamics on engaging partners to facilitate community empowerment for advocacy and change, rather than just as a means to improve research [26]. This may require reconceptualizing engagement from “sources of information” to “agents of change” [42] i.e., working further along the spectrum of community engagement [10,43].

Finally, our results clarify the challenges of doing PSM well within academic structures. Key facilitators of PSM discussed by participants include trusting relationships with partners, conducting practice-driven research, and working within diverse teams. Engaging in building trust with communities requires dedicated time and funding [44]. Grant timelines and academic incentives such as promotion and tenure can make taking the time to build these relationships with communities difficult, but institutional commitments to community engaged research can drive higher engagement from partners [45]. Conducting truly practice-driven research can also be difficult when academic researchers are typically funded to study a specific topic that must be decided upon a priori. Diverse teams require a supply of individuals trained in and committed to these methods, and institutional structures that support and reward team science.

Our study takes important steps towards understanding the unique benefits, challenges, and future needs for research integrating PSM and implementation research. Contrasted with prior literature reviews and commentaries, we collect primary data by speaking directly with researchers and practitioners who have experience with PSM approaches. Our sampling approach resulted in a substantial sample of researchers engaged in these methods across many institutions, but we were able to recruit comparatively fewer practitioners. Sometimes researchers were not comfortable introducing us to practice partners because these partners were already overburdened and busy, and sometimes there were concerns that their partners could not speak to our questions. Our results, particularly about benefits to practitioners, should therefore understood to draw primarily from an academic research perspective and transferability of practitioner-focused conclusions may be limited. Our interviews did not focus on specific methods and processes by which PSM was used in implementation research.

Moving forward, we offer several clear needs for the field of implementation science to consider in the support of rigorous, successful application of PSM and equitable, non-extractive engagement. It is critical to consider creative funding structures and streams to support the development of trusting researcher-practitioner partnerships. This is reflective of a larger issue in engaged research. Current funding approaches, including budgets and timelines, create structural barriers to building relationships because of limited funded time for researchers to deeply engage, long timelines to receive funding, and short funding periods [10,12]. These barriers are historic and will require systems change within academic institutions to overcome, which begins with a culture change from top leadership in these institutions [46].

Further, the range of skills needed to conduct this kind of research necessitates team science, which requires funding and institutional support of cross-disciplinary teams [47]. Training individuals in the skills for PSM projects necessitates ways to increase the pipeline of individuals who can facilitate participatory systems science work, for example through programs like the Systems Science for Social Impact training institute [48]. In working to build the evidence base for PSM projects, we also encourage researchers to consider qualitatively studying how engagement in research benefits (or does not benefit) partners, and developing ways to measure how partners benefit from engagement in PSM projects. It will also be important to study if and how PSM approaches improve implementation outcomes. Finally, we echo other calls for best practices focused on participatory processes and outcomes in implementation science [12]. Learnings from community-based system dynamics and the community-based participatory research field could provide helpful resources. Setting out best practices and standards for engaged research is critical to avoid reinforcing extractive or consultative engagement practices [12].

PSM approaches are well-suited to contribute to the goals of implementation science. Our findings can help strengthen the use of these methods by improving the field’s understanding of the unique benefits of engaging in the process of PSM itself and facilitators of success. Using these methods requires careful attention so that they do not reproduce consultative engagement patterns or power imbalances. Moving forward, the field should effectively fund participatory and engaged team science projects, train implementation scientists in these methods, and consider best practices for partnered research.

## Supporting information

10.1017/cts.2026.10692.sm001 Smith et al. supplementary material Smith et al. supplementary material

## Supplementary material

The supplementary material for this article can be found at https://doi.org/10.1017/cts.2026.10692.
