# Supplementary material for: Participatory systems modeling in implementation research: Exploring benefits, facilitators, and future needs
Source: J Clin Transl Sci. 2026 Feb 4;10(1):e48. doi: 10.1017/cts.2026.10692 (PMC12975621; doi:10.1017/cts.2026.10692)
Supplement: Smith et al. supplementary material [file S205986612610692Xsup001.pdf]

Document: Interview guide
Protocol title: Process benefits of participatory systems science methods for implementation research
Version: 3
Date: [fill in]

Getting started

Thank you for being here and scheduling an interview with me today. I'm excited to hear more about your work and experiences.

As you know, we are interested in hearing more from people like yourself about the potential benefits of engaging in the process of participatory systems science research – such as your work [tailored for subject]. Benefits of participatory systems science work are often mentioned in research, but we are worked to develop a more detailed and explicit understanding of these benefits in this study. Specifically, we're interested in learning what there is to gain from the process of using methods like group model building, agent-based modeling, system dynamics, or network analysis in collaboration with community partners, and what happens in these projects beyond the creation of a model.

Before we dive in, do you have any questions about your participation, or the consent form you received via email?

Great, so can I get your verbal consent to participate in this research project?

[provides verbal consent, recorded in research record]

Thank you, and are you ok with me recording this session? We will delete the recording once the interview transcript has been verified.

[says yes or no to recording]

[if no to recording]: That's fine, please just bear with me as I take notes during the conversation.

Recording begins

Interview

Let's start with hearing a bit about yourself. Can you give an overview of your background and how you came to be involved in research using participatory systems science research?

Could you tell us about the ***most recent*** participatory systems science project you've worked on? We would like to hear about the entire process you took.

Probes:

What was the objective of the project?

What specific systems science methods were used?

What types of individuals were on the project team (what types of researchers and practitioners) and how did you engage them?

When were those folks involved? At what point in the project were they involved?

What was their level of engagement? How were they involved? Who decided the level of involvement?

What was the timeline of the project?

Note to interviewer: think about whether this project is mapping, modeling, or use.

Reflecting back on this process, can you talk about the benefits of the ***participatory nature*** of the project?

And what are the benefits of the ***systems science nature*** of the project?

Now thinking about them, in what we consider ***participatory systems science***, what are the benefits of that integrated process?

Probes for each aspect:

What do you think were the benefits for researchers?

What do you think were the benefits for practitioners and/or community, those on the ground of implementation?

What things made it possible (easier?) for those benefits to come about?

What things made it or could have made it harder to get to those benefits?

Would reflecting back on other projects add or change any of your thoughts? If so, how?

Probes:

Ask about mapping, modeling, and use in past projects if they've only talked about one.

While we've been asking about the benefits of participatory work, it's clear that the process also requires time and resources *[tailored to what they've said above]*. Can you talk a bit about the difficulties of engaging in participatory systems science work, and tradeoffs between these difficulties and benefits you've discussed? *[inclusion of this question will depend on answers to prior questions & initial use of interview guide with participants]*

Thinking about your future work, is there anything you would change about your approach to participatory approaches to systems science work?

What is needed to move the field forward so that more projects can use participatory systems science methods and get the benefits you described?

Probes:

Ideas for products created by researchers?

Strategies for engaging partners?

Changes to research infrastructure (funding, longer project timeframes)?

[Summarize what they've told us]

In our last few minutes, what haven't we asked about that you think is important for us to know and communicate as we think through our results?

And as a final question, do you have suggestions for folks who are outside of academic and more practice-focused that might be good for us to talk to?

Thank you so much for taking the time to talk with me today. As a thank you for participating, we'd like to send you a $50 Amazon gift card; is the email you signed up for this interview with the appropriate one to use?

Node	Code Name	Description and examples	Corresponding Interview Question(s)
1.0	Background of interviewee and project		Can you give an overview of your background and how you came to be involved in research using systems science methods?
1.1	Description of project		Could you tell us about a participatory systems science project you worked on somewhat recently?
1.2	Project objective		What was the objective of the project?
1.3	Systems science method(s) used	This should be used to capture discussion about the *specific kinds of methods used* – for example, causal loop diagramming, system dynamics, agent-based modeling, network analysis. Can also capture methods that are adjacent and/or used to get inputs for the systems science methods, such as surveys or qualitative interviews.	What specific systems science methods were used?
1.4	Who was engaged	This should capture the individuals who were engaged as part of the participatory project (community members, coalition leaders, policymakers). We'll use this to characterize the full breadth of folks engaged.	What types of individuals were on the project team (what types of researchers and practitioners) and how did you engage them?
1.5	Purpose of engagement	This should capture *why* participatory approaches were used in the project. Some of this may be overlapping with the project objective, but what we are looking for here is the information to capture the full spectrum of engagement, from things like 'mapping the full system' to 'using the final agent-based model'.	
2.0	Benefits of using participatory systems science		Reflecting on this process, can you talk about the benefits you observed?
2.1	Benefits to researchers	All large chunks of text where respondents discussed their perceived benefits of participatory systems science work *to researcher/projects*.	What do you think were the benefits for researchers?

		Note: we will later use these responses to identify different themes of benefits to researchers.	
2.2	Benefits to implementers	All large chunks of text where respondents discussed their perceived benefits of participatory systems science work to those that were involved in the project – whether that be community members, implementation leadership, or policymakers.<br><br>Note that this discussion of benefit will vary based on who was involved. For community members, what was the benefit to them of participating? For policy makers, what was their benefit?	What do you think were the benefits for practitioners, those on the ground of implementation?
3.0	Facilitators of participatory systems science	Things that respondents talked about that made their work easier. Some examples include the intuitiveness of systems science methods (people do inherently understand complexity) or prior engagement and relationships with folks.	What things made it possible (easier?) for those benefits to come about?
4.0	Challenges and barriers to participatory systems science	This captures the variety of difficulties that folks talked about that made their work more difficult. In initial coding, it was often difficult to distinguish between barriers to getting the benefits of participatory systems science, and challenges to systems science methods in general. We opted to combine them and perhaps parse out differences later on when we look for broader themes.<br><br>Examples include the time and effort required for high-quality, sustained engagement, data needs, buy-in, or participant understanding.	What things made it or could have made it harder to get to those benefits?<br><br>Can you talk a bit about the difficulties of engaging in participatory systems science work, and tradeoffs between these difficulties and benefits you've discussed?
5.0	What is needed to advance participatory systems science		What is needed to move the field forward so that more projects can use participatory systems science methods and get the benefits you described?
5.1	Ideas for products needed		Ideas for products created by researchers?
5.2	Ideas for engagement strategies		Strategies for engaging partners?

5.3	Ideas for changing research infrastructure		Changes to research infrastructure (funding, longer project timeframes)
6.0	Other things important to know	Responses varied widely here – and often were related to previous interview questions where the respondents went back and talked about other facilitators, barriers, products, etc. This may not end up being a useful standalone quote and instead most answers here might be absorbed into other codes.	In our last few minutes, what haven't we asked about that you think is important for us to know and communicate as we think through our results?
7.0	Good quotes	Used to pull out nice phrases that we want to use to highlight specific themes.	n/a
8.0	New ideas	Use as needed for things that are interesting/possibly useful for future work and ideas, but not relevant to our goals here.	n/a

Default Question Block

Thanks for your time contributing to our study thus far. To provide context for our findings, we are asking participants to fill out a short survey collecting some basic demographic and contextual information. Your answers to this survey will be kept strictly confidential and will not be linked to interview data, and we will only report aggregate results. This should take less than 5 minutes to complete.

This study has been approved by Harvard (IRB22-1419) and Washington University in St. Louis (202006180). If you have any questions you may contact the PI's, Natalie Smith (nataliesmith@hsph.harvard.edu) and Stephanie Mazzucca-Ragan (smazzucca@wustl.edu).

By clicking the arrow below to continue with the survey, you agree to be a part of this research study.

What is your age in years? Please enter the closest whole number.

____

What is your gender?

- ○ Female
- ○ Male
- ○ Non-binary / third gender
- ○ Prefer not to answer

How would you classify your race? Please check all that apply.

- ☐ White
- ☐ Black
- ☐ Asian
- ☐ Native Hawaiian or Pacific Islander
- ☐ Native American or Alaskan Native
- ☐ Another group
- ☐ Don't know
- ☐ Prefer not to answer

Would you consider yourself of Latino or Hispanic ethnicity?

- ◯ Yes
- ◯ No
- ◯ Prefer not to answer

What is your highest level of education?

- ◯ High school degree
- ◯ Some college
- ◯ Bachelor's (BS, BA, and similar)
- ◯ Master's (MBA, MS, MPH, MSW, and similar)
- ◯ PhD
- ◯ MD or other professional degree
- ◯ Prefer not to answer

What is your main area of study/work?

____

Would you consider your work more research or practice focused?

All Research Focused   All Practice Focused

How many years have you been working in your field? Please enter the closest whole number.

_____

Do you consider yourself a community engaged researcher? By community engaged research, we mean research that involves purposeful collaboration between researchers and community members.

- ◯ Yes
- ◯ No
- ◯ Prefer not to answer

What systems science methods have you used in your work? Please check all that apply.

- ☐ Social network analysis
- ☐ Causal loop diagramming (inclusive of group model building efforts)
- ☐ System dynamics
- ☐ Agent-based modeling
- ☐ ________ Other (please specify)

Powered by Qualtrics
